# Supplementary material for: Role of atmospheric rivers in shaping long term Arctic moisture variability
Source: Nat Commun. 2024 Jun 29;15:5505. doi: 10.1038/s41467-024-49857-y (PMC11217282; doi:10.1038/s41467-024-49857-y)
Supplement: Supplementary file 1 — Supplementary Information [file 41467_2024_49857_MOESM1_ESM.pdf]

Supplementary information for

Role of atmospheric rivers in shaping long term Arctic moisture variability

Zhibiao Wang[1], Qinghua Ding[2*], Renguang Wu[3], Thomas J. Ballinger[4*], Bin Guan[5], Deniz Bozkurt[6], Deanna Nash[7], Ian Baxter[2], Dániel Topál[8], Zhe Li[2], Gang Huang[9], Wen Chen[10], Shangfeng Chen[1], Xi Cao[1], Zhang Chen[11]

[1]*Center for Monsoon System Research, Institute of Atmospheric Physics, Chinese Academy of Sciences, Beijing, China*

[2]*Department of Geography, and Earth Research Institute, University of California, Santa Barbara, Santa Barbara, California, USA*

[3]*School of Earth Sciences, Zhejiang University, Hangzhou, China*

[4]*International Arctic Research Center, University of Alaska Fairbanks, Fairbanks, AK, USA*

[5]*Joint Institute for Regional Earth System Science and Engineering, University of California, Los Angeles, Los Angeles, California, USA*

[6]*Department of Meteorology, University of Valparaíso, Chile, Center for Climate and Resilience Research (CR)2, Chile, Center for Oceanographic Research COPAS COASTAL, University of Concepción, Chile*

[7]*Center for Western Weather and Water Extremes, Scripps Institution of Oceanography, University of California San Diego, La Jolla, California, USA*

[8]*Earth and Climate Research, Earth and Life Institute, Université catholique de Louvain, Louvain-la-Neuve, Belgium*

[9]*State Key Laboratory of Numerical Modelling for Atmospheric Sciences and Geophysical Fluid Dynamics, Institute of Atmospheric Physics, Chinese Academy of Sciences, Beijing, China*

[10] *Department of Atmospheric Sciences, Yunnan University, Kunming, China*

[11] *School of Atmospheric Sciences, Chengdu University of Information Technology, Chengdu, China*

*Corresponding authors: Qinghua Ding, Department of Geography, University of California Santa Barbara, Santa Barbara, California, USA and Thomas J. Ballinger, International Arctic Research Center, University of Alaska Fairbanks, Fairbanks, AK, USA

E-mail: qinghua@ucsb.edu and tjballinger@alaska.edu

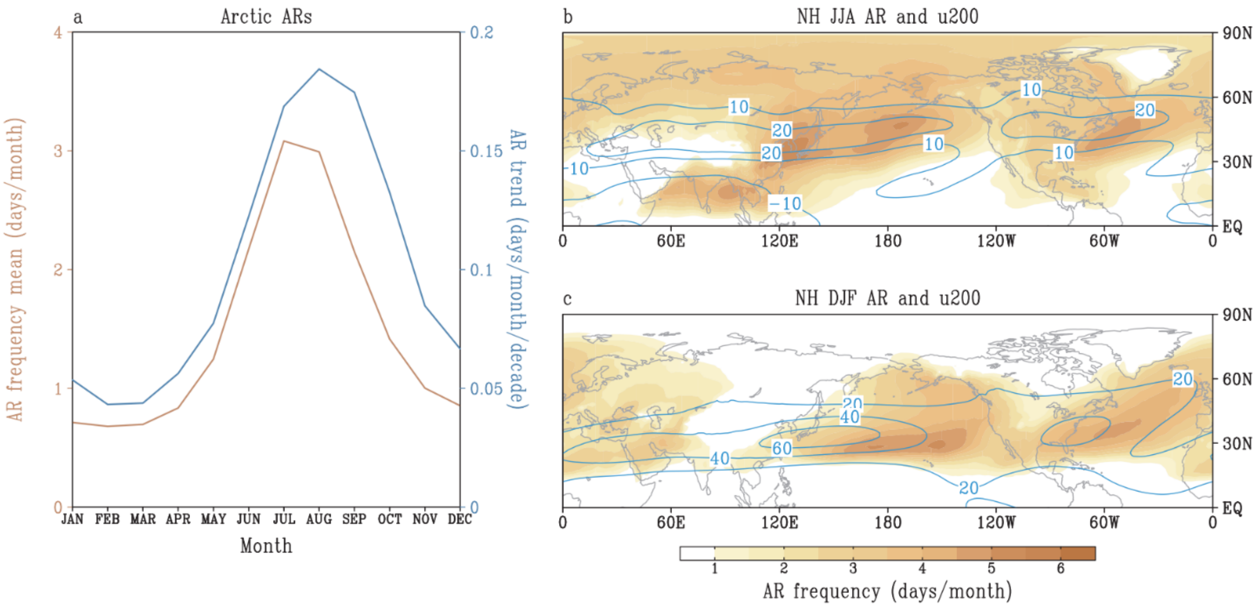

Supplementary Figure 1. a, Climatological monthly mean AR frequency (unit: days/month, brown line) and their trends (unit: days/month/decade, blue line) within the Arctic (north of 60°N) from January to December based on ERA5 reanalysis for the period 1979-2019. **b-c**, Climatological summer (JJA) (**b**) and winter (DJF) (**c**) AR frequency (shading, unit: days/month) and zonal winds at 200hPa (contour, unit: m/s, summer interval: 10 m/s, winter interval: 20 m/s) in the Northern Hemisphere from 1979 to 2019.

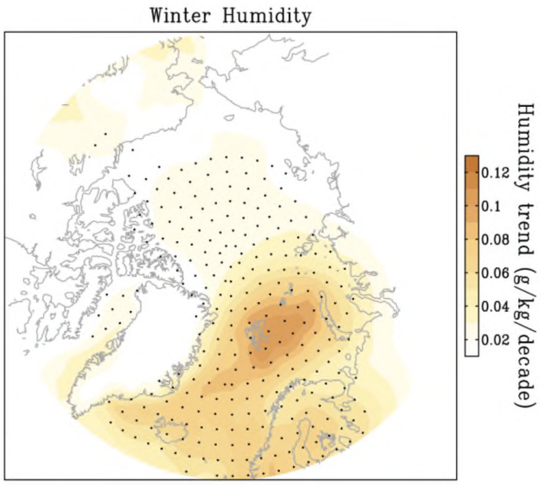

Supplementary Figure 2. Linear trends of winter lower to middle tropospheric (surface to 500 hPa average) specific humidity (g/kg/decade) in the Arctic from 1979 to 2019. Black dots denote statistically significant trends at the 95% confidence level.

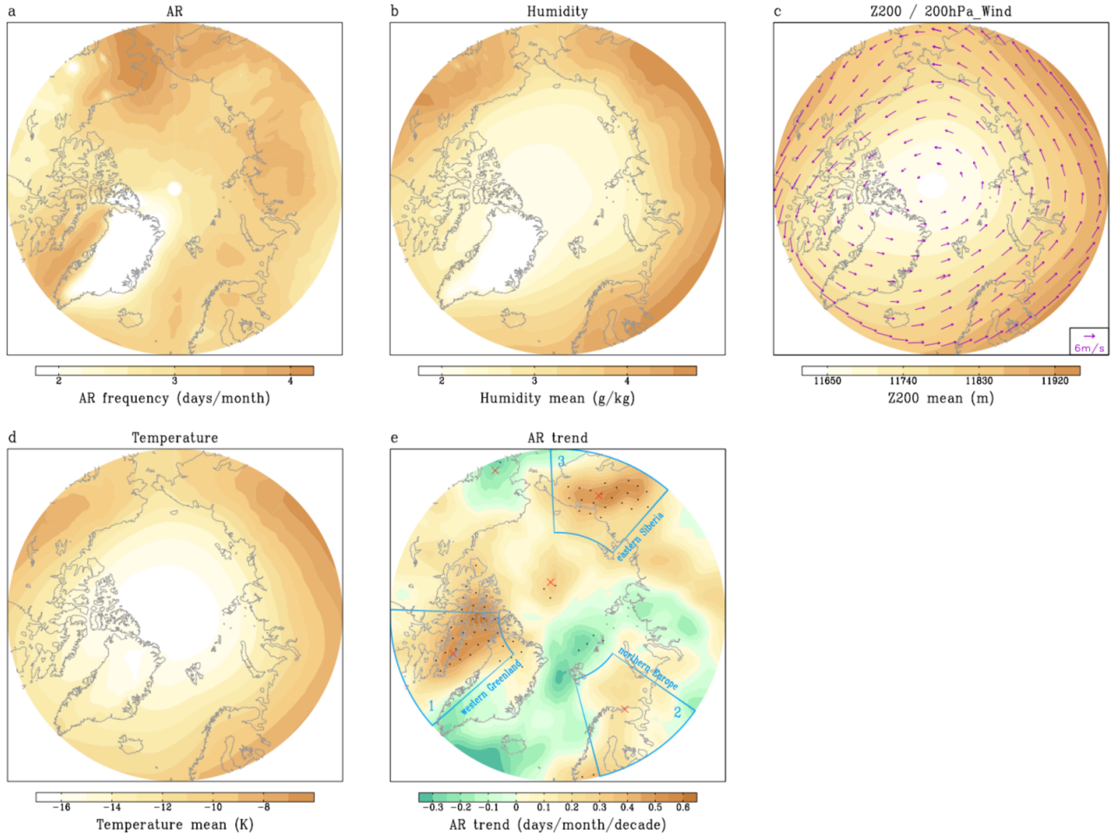

Supplementary Figure 3. Climatological summer (JJA) AR frequency (**a**, unit: days/month), lower to middle tropospheric (surface to 500 hPa average) specific humidity (**b**, unit: g/kg), 200 hPa geopotential height (Z200) (**c**, unit: m), tropospheric (surface to 200 hPa average) air temperature (**d**, unit: K), and linear trends of ARs (**e**, unit: days/month/decade) in the Arctic from 1979 to 2019. **e** The same as Figure **1a**, but including the three key regions (blue fan-shaped covered area), marked as western Greenland (1), northern Europe (2), and eastern Siberia (3), respectively, with central points of the three key regions, as well as locations near the North Pole and southwestern Alaska, indicated by red crosses.

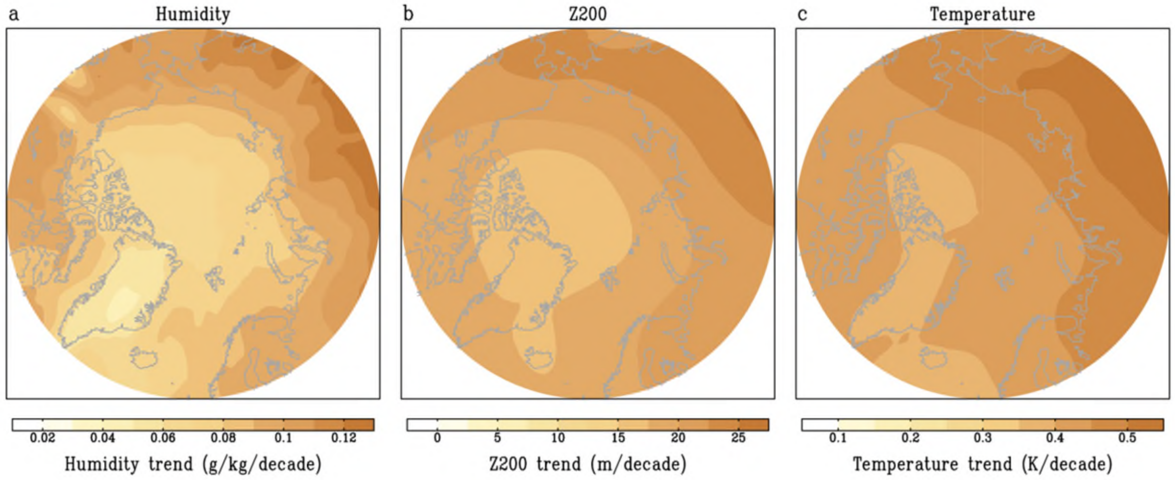

Supplementary Figure 4. Linear trends of summertime lower to middle tropospheric (surface to 500 hPa average) specific humidity (g/kg/decade) (**a**), 200 hPa geopotential height (Z200) (m/decade) (**b**), and tropospheric (surface to 200 hPa average) air temperature (K/decade) (**c**) in the Arctic from 1979 to 2014 based on 34 CMIP6 models. Trends of these three variables in **a-c** are significant everywhere at the 95% confidence level.

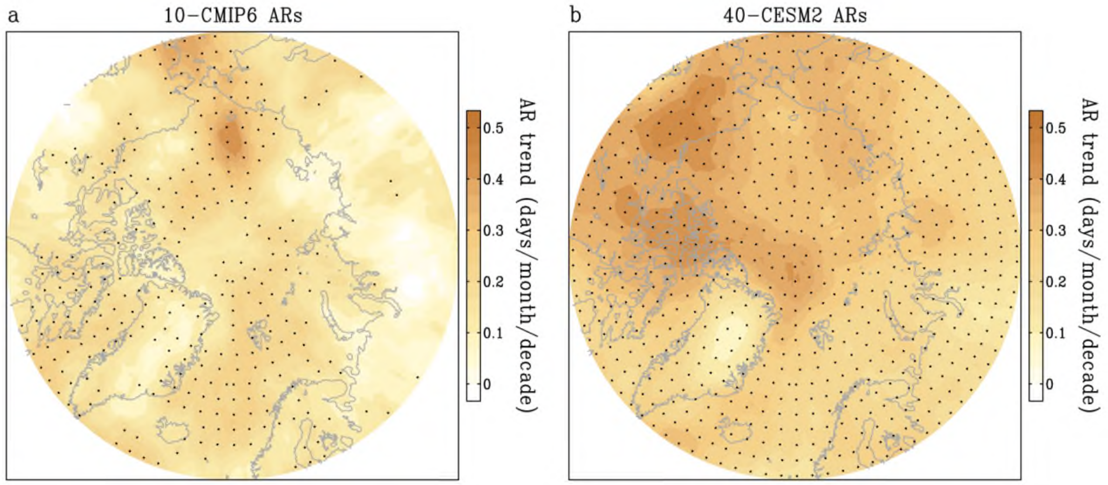

Supplementary Figure 5. Linear trends of summer (JJA) AR frequency (days/month/decade) in the Arctic based on (**a**) 10 CMIP6 (10-CMIP6) models from 1979 to 2014 and (**b**) 40-member CESM2 simulations (CESM2-LEN) from 1979 to 2019. Black dots in **a**-**b** denote statistically significant trends at the 95% confidence level.

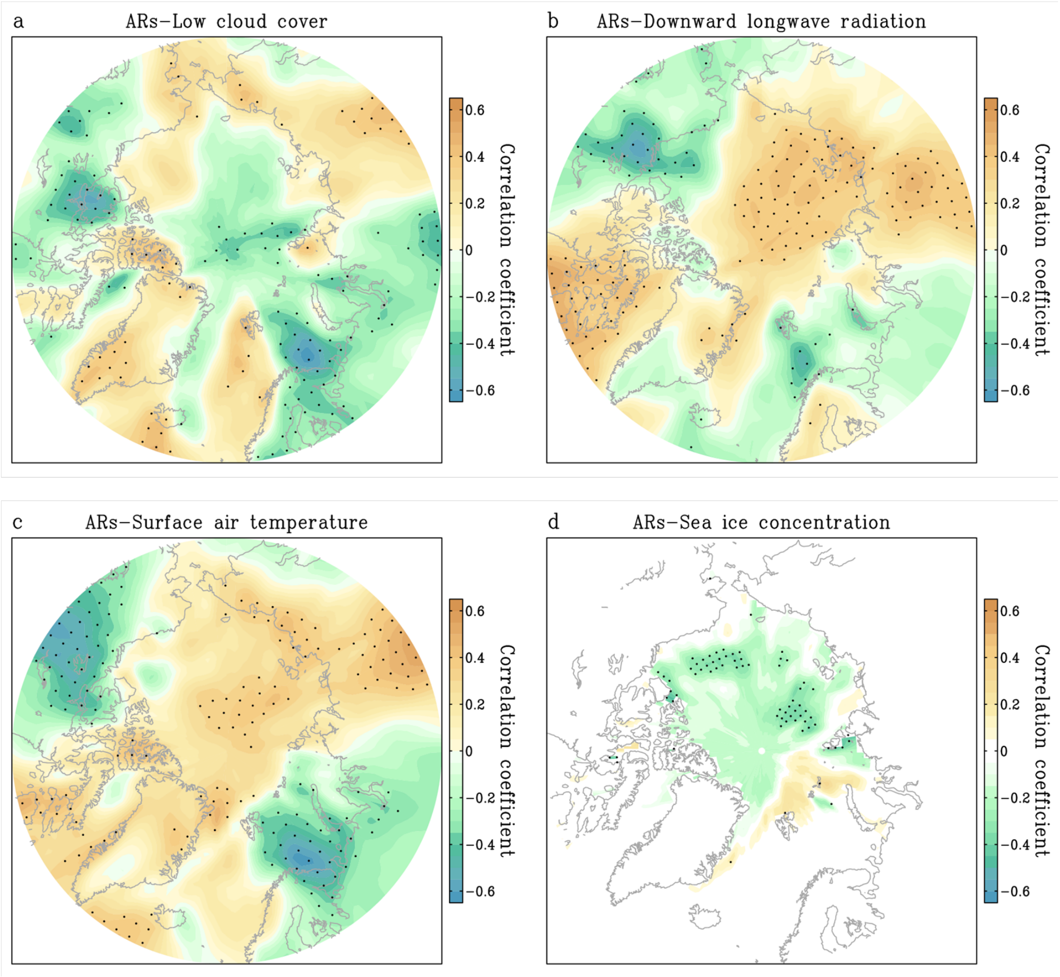

Supplementary Figure 6. Correlation coefficients between the leading MCA-AR time series and low cloud cover in summer (JJA) (**a**), downward longwave radiation in summer (**b**), surface air temperature in summer (**c**), and sea ice concentration in September (**d**) in the Arctic from 1979 to 2019. Black dots in **a-d** denote statistically significant correlations at the 95% confidence level.

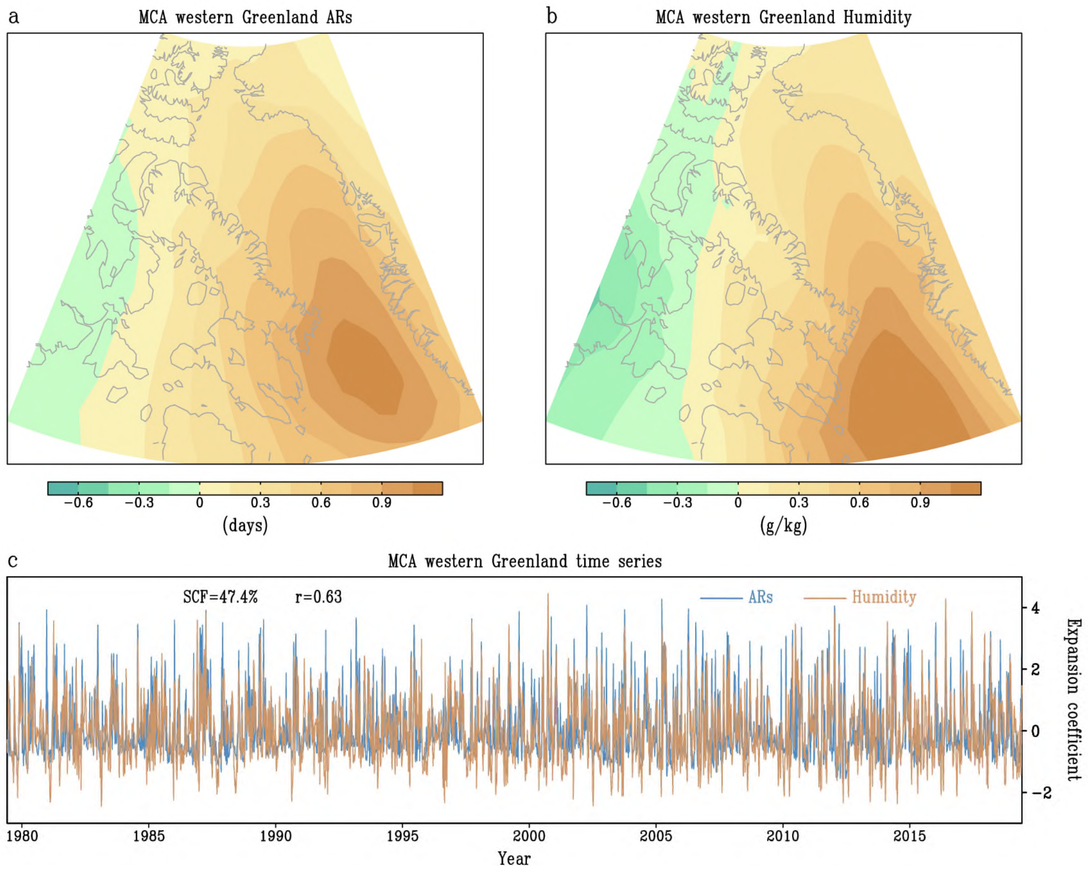

Supplementary Figure 7. a-b, Spatial patterns of the leading MCA mode between daily AR freqnecy (**a**, in days) and lower to middle tropospheric specific humidity (**b**, in g/kg) over western Greenland in summer during 1979-2019 (detrended). **c**, Standardized time series of the two pattens (Z200 and ARs) in the leading MCA mode from 1979 to 2019. 'SCF' in **c** indicates the squared covariance fraction of the leading MCA mode. 'r' in **c** indicates the correlation coefficient between the time series of the two patterns in the leading MCA mode.

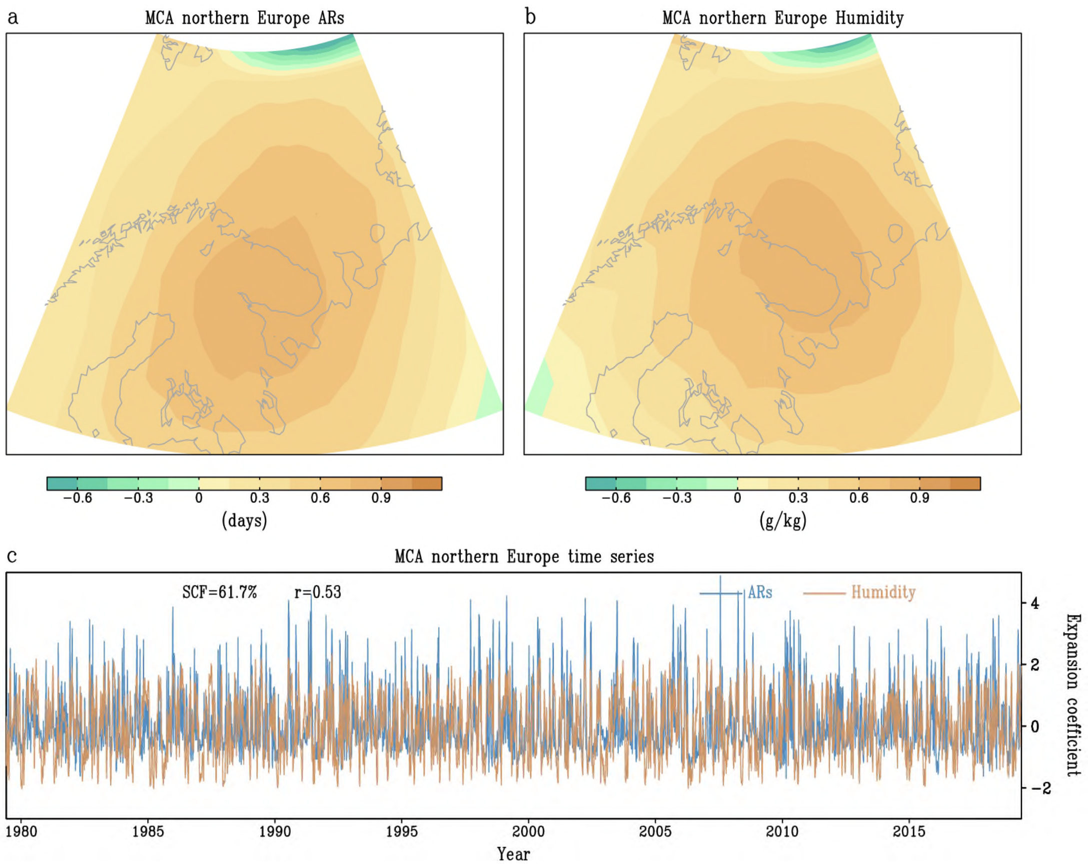

Supplementary Figure 8. The same as Supplementary Figure 7, but for the northern Europe region.

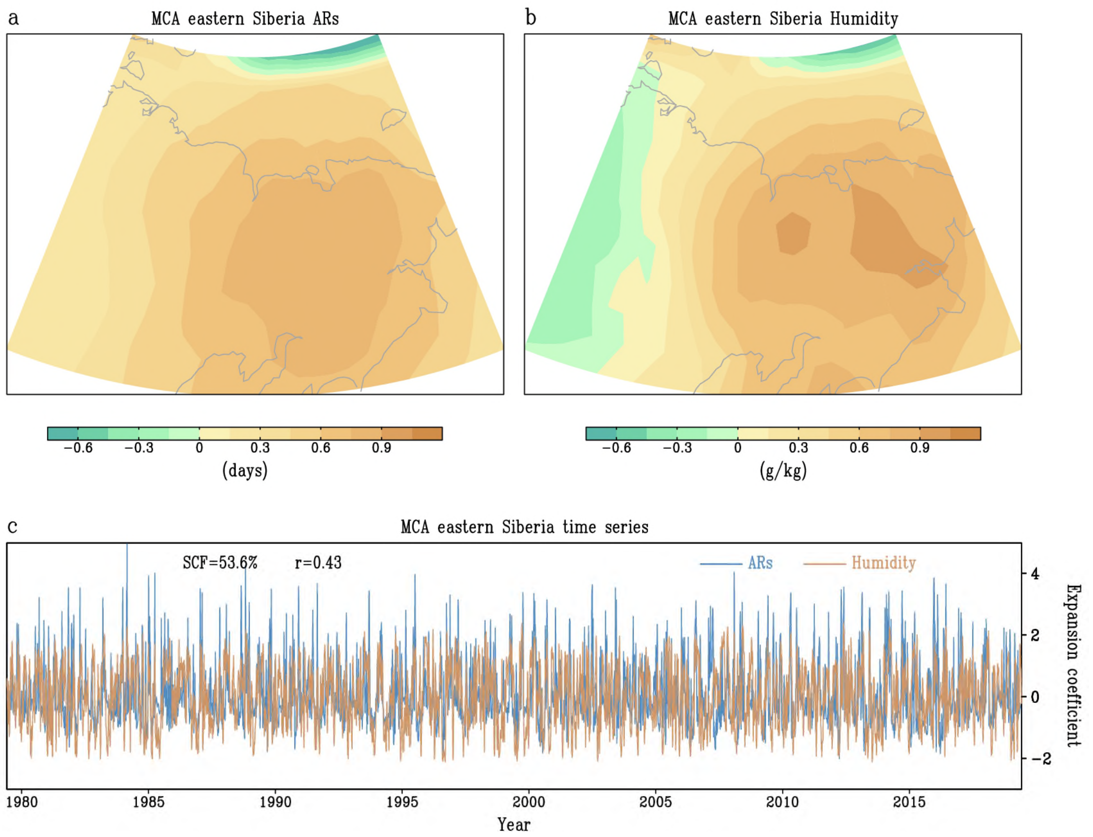

Supplementary Figure 9. The same as Supplementary Figure 7, but for the eastern Siberia region.

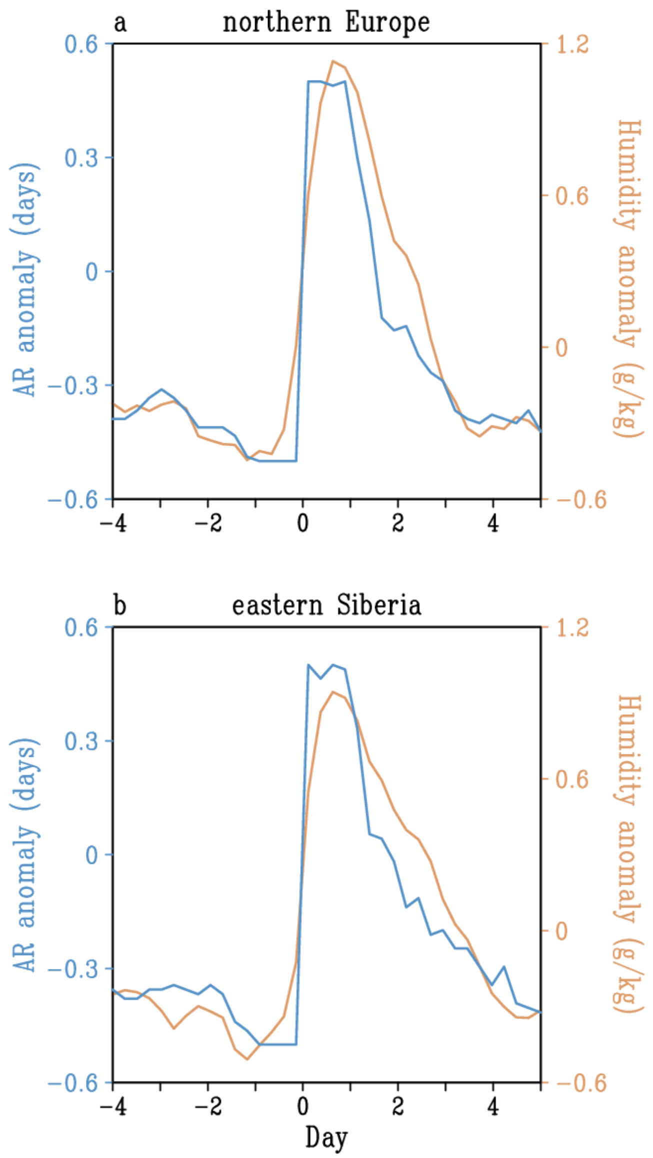

Supplementary Figure 10. a-b, Composite 6-hourly AR (days) and lower to middle tropospheric specific humidity (g/kg) anomalies (from four days before to five days after the outbreak of ARs) within northern Europe (**a**) and eastern Siberia (**b**) regions from 1979 to 2019 (detrended and with climatological seasonal cycles removed).

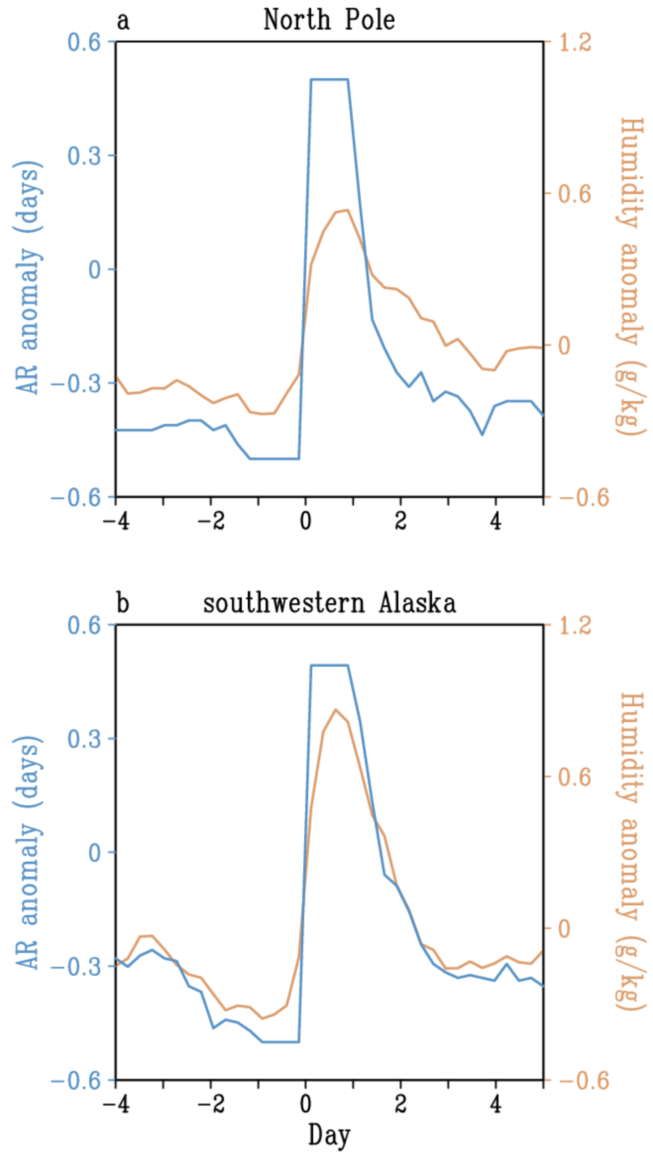

Supplementary Figure 11. The same as Supplementary Figure 10, but for areas near the North Pole (**a**) and southwestern Alaska (**b**).

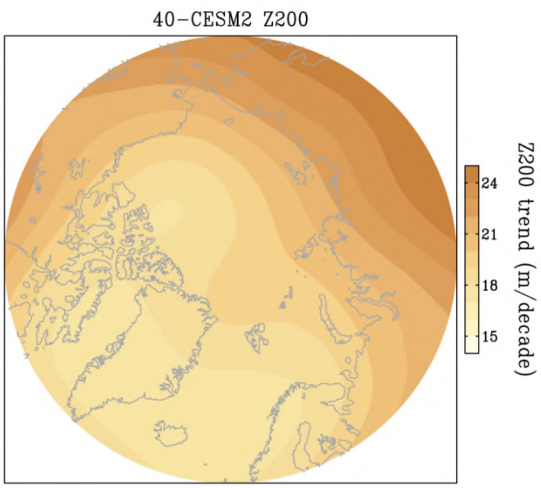

Supplementary Figure 12. Linear trends of summer (JJA) Z200 (m/decade) in the Arctic based on 40-member CESM2 simulations (CESM2-LEN) from 1979 to 2019. The trends of Z200 are significant everywhere at the 95% confidence level.

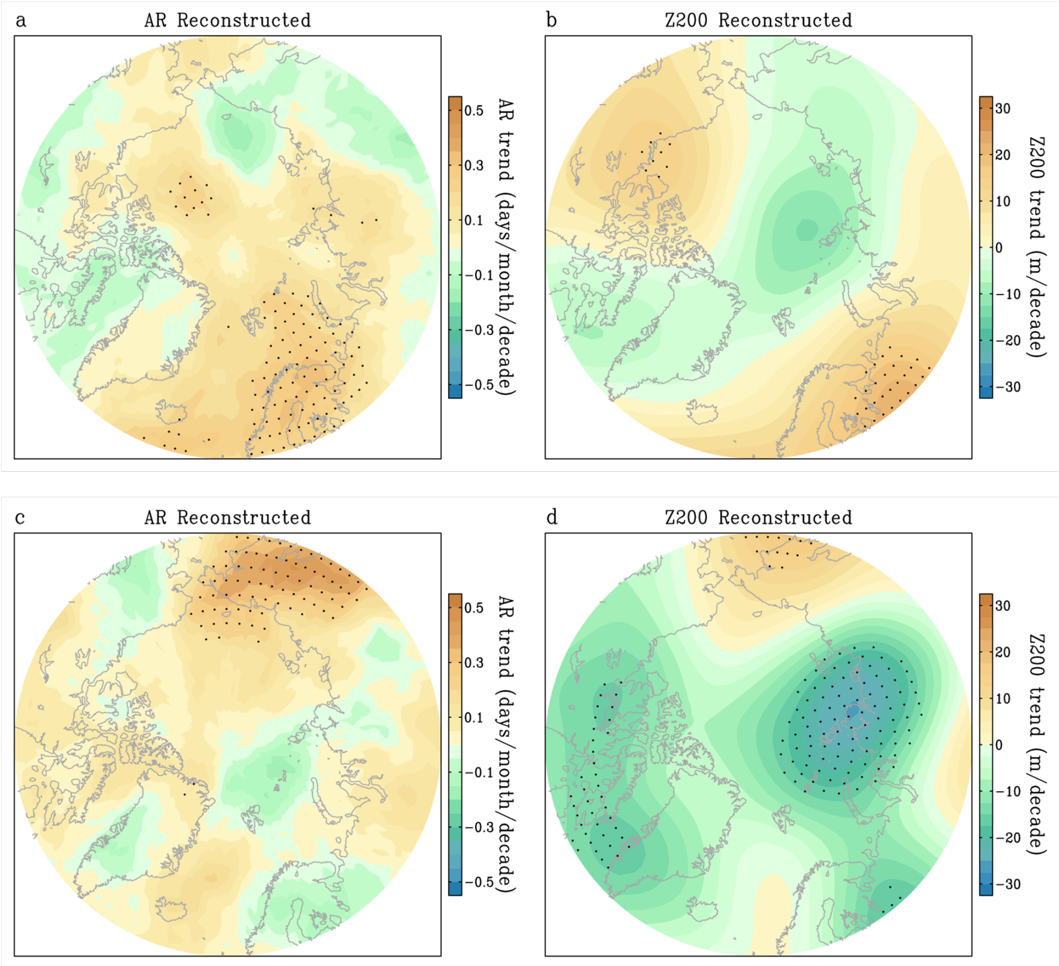

Supplementary Figure 13. Linear trends of reconstructed summer AR frequency (days/month/decade) (**a, c**) and Z200 (m/decade) (**b, d**) derived from the subgroups exhibiting strong increasing AR frequency trends over northern Europe (8 members) and eastern Siberia (8 members), respectively. The 40 members from CESM2-LEN are used in this calculation (see Methods). Black dots denote statistically significant trends at the 95% confidence level.

Supplementary Table 1. 34 climate models in the CMIP6 historical run. The table lists 34 CMIP6 models used to investigate the response of specific humidity, geopotential height, and air temperature to anthropogenic forcing in the study. The spatial resolution of each model is also provided.

CMIP6 model	Resolution (Lon × Lat)	CMIP6 model	Resolution (Lon × Lat)
ACCESS-CM2	1.875° × 1.25°	GISS-E2-1-G-CC	2.5° × 2°
ACCESS-ESM1-5	1.875° × 1.25°	GISS-E2-1-H	2.5° × 2°
BCC-CSM2-MR	1.12° × 1.12°	INM-CM4-8	2° × 1.5°
BCC-ESM1	2.8125° × 2.8125°	INM-CM5-0	2° × 1.5°
CAMS-CSM1-0	1.125° × 1.125°	IPSL-CM6A-LR	2.5° × 1.25°
CESM2	1.25° × 0.9°	KACE-1-0-G	1.3° × 0.9°
CESM2-FV2	2.5° × 1.9°	MCM-UA-1-0	3.75° × 2.24°
CESM2-WACCM	1.25° × 0.9°	MIROC6	1.40625° × 1.40625°
CESM2-WACCM-FV2	2.5° × 1.9°	MPI-ESM-1-2-HAM	1.9° × 1.9°
CIESM	1.25° × 0.9375°	MPI-ESM1-2-LR	1.9° × 1.9°
CanESM5	2.8125° × 2.8125°	MRI-ESM2-0	1.125° × 1.125°
E3SM-1-0	1.25° × 0.9375°	NESM3	1.875° × 1.875°
E3SM-1-1	1.25° × 0.9375°	NorCPM1	2.5° × 1.875°
FGOALS-f3-L	1.25° × 1°	NorESM2-LM	2.5° × 1.89°
FGOALS-g3	2° × 2.25°	NorESM2-MM	2.5° × 1.89°
GFDL-ESM4	1° × 1°	SAM0-UNICON	1.25° × 0.9375°
GISS-E2-1-G	2.5° × 2°	TaiESM1	1.25° × 0.9°

Supplementary Table 2. 10 climate models in the CMIP6 historical run. The table lists 10 CMIP6 models used to investigate the response of ARs to anthropogenic forcing in the study. The spatial resolution of each model is also provided.

CMIP6 model	Resolution (Lon × Lat)
BCC-ESM1	2.8125° × 2.8125°
CESM2-FV2	2.5° × 1.9°
CESM2-WACCM	1.25° × 0.9°
CESM2	1.25° × 0.9°
FGOALS-g3	2° × 2.25°
GISS-E2-1-G	2.5° × 2°
IITM-ESM	1.875° × 1.915°
MIROC6	1.40625° × 1.40625°
MRI-ESM2-0	1.125° × 1.125°
NESM3	1.875° × 1.875°
